# Ecological Momentary Assessment for Assessing Affect Patterns Associated With Depression in Cancer Survivors in Primary Care: A Pilot Study

**DOI:** 10.1002/pon.70367

**Published:** 2025-12-31

**Authors:** Jolien A(lissa). Panjer, Mariken E(lisabeth). Stegmann, Daan Brandenbarg, Maya J. Schroevers, Harriëtte Riese, Evelien Snippe, Huibert Burger

**Affiliations:** ^1^ Department of Primary and Long‐Term Care, Cure and Care in the Community Context (FOUR‐C) ‐ Research Program University of Groningen University Medical Center Groningen Groningen the Netherlands; ^2^ Department of Health Sciences Section Health Psychology University of Groningen University Medical Center Groningen University Medical Center Groningen Groningen the Netherlands; ^3^ Department of Psychiatry, Clinical Cognitive Neuropsychiatry Research Program (CCNP) University of Groningen University Medical Center Groningen Groningen the Netherlands; ^4^ Department of Developmental Psychology University of Groningen Groningen the Netherlands

**Keywords:** cancer survivors, depression, ecological momentary assessment, feasibility, primary care, psycho‐oncology

## Abstract

**Introduction:**

Depressive symptoms are common in cancer survivors. Recognizing depression can be complicated due to recall bias or oncological treatment‐related symptoms including cognitive problems, which in turn may undermine the reliability of self‐report questionnaires.

**Aim:**

To explore the feasibility and patient satisfaction of smartphone‐based Ecological Momentary Assessment (EMA) in primary care cancer survivors.

**Methods:**

Patients > 18 years, curatively treated for cancer within the past two years, regardless whether they experienced depressive symptoms, were selected based on the GPs' health records. EMA questionnaires were sent three times daily for 6 weeks, covering positive and negative affect, along with related experiences. Patients received weekly EMA feedback reports. After the EMA period, they completed an evaluation questionnaire and participated in a follow‐up phone call to discuss their EMA experiences.

**Results:**

Patient recruitment achieved a reach of 17.0% who were invited for participation (158/931), of whom 33/158 agreed to participate yielding a response rate of 20.9%. Patients found the EMA questions clear and study participation easy with a completion rate of 67% among those who started. However, 64% felt the frequency of EMA prompts was excessive, with 52% considering the 6‐week duration appropriate and 48% feeling it was too long. During phone call evaluations, patients reported becoming inattentive with filling out the EMA's. Weekly reports were viewed as relevant and provided valuable insights into levels and changes in their mood.

**Conclusion:**

The relatively low reach and response rate do not entirely support the feasibility and acceptability of a 6‐week EMA in cancer survivors in primary care without depressive symptoms. EMA was, however, completed by a majority among those who started and was regarded as a user‐friendly tool that offered valuable insights to individuals. It could potentially benefit cancer survivors or other patients who do experience depressive symptoms in primary care.

## 1 Background

The number of cancer survivors is expected to rise in the coming years [1] due to aging of the population and improved staging and treatments [2]. Consequently, an increasing number of patients are facing long‐term effects, including depression. This affects 17% of patients 5–10 years after diagnosis, from a cohort with mixed tumor entities [1], profoundly impacting personal, social and work life [3, 4] and reducing quality of life [5].

Early recognition of depressive symptoms is important because this, if followed by adequate psychological care, can prevent depression [6]. In many countries, including the Netherlands, early recognition is a task for primary care. However, early recognition is challenging, for both general practitioners (GPs) and patients. It has been reported that GPs did not recognize depression in over 60% of patients [7]. A significant cause for this is that the anamnesis or the completion of a questionnaire largely relies on a person's memory [8]. Specifically in cases of depression, memory and concentration problems are common, increasing the likelihood of recall bias [9]. In cancer survivors, this may be amplified by cognitive dysfunction, often linked to cytostatics [10] and endocrine therapy for hormone sensitive breast cancer [11], with incidence rates until 2 years after treatment reported to be between 19% and 78% [12]. Additionally, the mood congruency effect: being in a positive mood state when consulting the GP, makes it harder to remember depressive symptoms [13].

An alternative method for timely detecting depressive symptoms, by detecting daily life experiences associated with the risk of developing depressive symptoms, is Ecological Momentary Assessment (EMA). EMA may alleviate the aforementioned issues related to recall bias and the mood congruency effect [4, 14]. This method is based on sending short, typical digital, questionnaires on momentary experiences multiple times a day during multiple days or weeks, thereby reducing the risk of recall bias and the mood congruency effect [15, 16]. By using EMA, for example the presence of negative affect and absence or lower rates of positive emotions in daily life can be detected, which are core elements related to depression [17]. The results from the EMA questionnaires can be used in consultation when discussing possible depressive symptoms.

Over the last years, EMA has been used for investigating fatigue, chronic illnesses and psychiatric illnesses [18, 19, 20, 21, 22]. Several studies tested EMA within the community and primary care; the feasibility has been tested for chronic diseases and for detecting suicide risk, with mixed results [19, 23].

Among cancer survivors, EMA has been used to investigate fatigue and exercise [24, 25, 26]. These studies showed that EMA was feasible and useful for monitoring daily life experiences associated with depression and fatigue in patients treated in secondary care, that is receiving treatment from specialist services or mental health institutions upon referral from primary care. However, following cancer treatment, most patients transition from secondary to primary care [27]. In primary care symptoms may be less severe and impactful on daily life, potentially reducing motivation and feasibility of EMA. Consequently, before investigating the effectiveness of EMA in primary care, more knowledge on its feasibility in this particular setting and with this particular population is needed. To our knowledge, there are no studies on the feasibility of EMA for assessing mood symptoms in cancer survivors in primary care.

This pilot study therefore aims to assess the feasibility and patient satisfaction of smartphone‐based EMA for getting insight in the affect patterns related to depression in cancer survivors in primary care. These results can be discussed in consultation with healthcare professionals, when discussing emotions experienced by cancer survivors. The focus of this study is primarily on selection and inclusion of patients, adherence to EMA assessment and patient satisfaction. Secondly, we will assess levels of measured positive and negative affective experiences for descriptive purposes and discuss the potential research and clinical applications of EMA in general practice.

## 2 Methods

### 2.1 Study Design

This pilot study focused at the assessment of feasibility of using EMA among patients from general practices in the Northern part of the Netherlands. The Medical Research Ethics Committee of the University Medical Center Groningen, the Netherlands, concluded that this study was not subject to the Dutch Medical Research Involving Human Subjects Act (registration number: 20200464). All participants gave written or digital informed consent to take part in the study.

### 2.2 Study Population

Patients were eligible for participating when they met the following inclusion criteria: age ≥ 18 years at time of invitation, treated with curative intent for a malignancy and initial treatment finished 1 month to maximum 2 years before time of invitation. Stage of the malignancy was not taken into account. Initial treatment referred to the treatment plan for curation of the malignancy, meaning: surgery, chemotherapy, radiotherapy, similar treatment or a combination (excluding hormonal therapy for several years or a similar new therapy).

Patients were not eligible when they met one, or more, of the following exclusion criteria: major depressive disorder requiring immediate treatment, for example in case of suicidality, presently receiving treatment outside GP practice for depressive symptoms/depressive disorder at time of inclusion, not able to fill out questionnaires (e.g.,: severe cognitive symptoms, not sufficiently skilled in Dutch language) or objection from GP for inviting the patient for any reason. Patients receiving treatment for depressive symptoms were excluded, to create a homogeneous sample focused on identifying new patients at risk for depression.

### 2.3 Recruitment of Practices

GP practices were recruited through the Academic General Practitioner Development Network (in Dutch: Academisch Huisarts Ontwikkel Netwerk, AHON),in the Northern part of the Netherlands. These practices were asked to participate in our study through a monthly newsletter, but none responded. Subsequently, 37 non‐responding practices were contacted by telephone, of which three agreed to participate. Eighteen practices were contacted through the researchers' personal network, of which thirteen participated. New practices were added until the target number of patients was reached. Patients were included between November 2020 until March 2022.

### 2.4 Recruitment of Participants

We aimed to include 30 patients. For patient identification, we searched the GP's Electronic Patient File (EPF) system based on the inclusion criteria. In Dutch GP's EPFs, codes are registered during each patient contact using the International Classification of Primary Care‐1 (ICPC). All contacts or administrative tasks of patients with the GP concerning the same diagnosis and/or symptoms are registered under the same code. We searched for patients using age ≥ 18 years and registration in EPF regarding cancer related ICPC codes in the past 2 years. Supporting Information S1: List S1 shows the ICPC codes for the malignancies of included patients. The GP was asked to confirm patient eligibility, and to record reasons for exclusion. The EPF search was repeated after three and 6 months. GPs sent all eligible patients an invitation letter, information about the study and an informed consent form. If patients agreed to participate, the EMA was started.

### 2.5 Study Procedures

Baseline and EMA questionnaires were filled out online using a secure online questionnaire application Roqua (www.roqua.nl). The EMA questionnaire could be filled out on an iPhone or Android smartphone. Patients without a smartphone were provided one for the study duration.

#### 2.5.1 Baseline Questionnaire Assessments

At the start of the study patients were asked to complete a baseline questionnaire which assessed demographics (age, gender, highest finished education level, social support) and employment (whether patients have a job, absenteeism). Patients were asked to fill in a depression questionnaire (inventory of depressive symptomatology (IDS)). This questionnaire comprehensively includes and operationalizes all DSM criteria for depressive mood disorders [28].

#### 2.5.2 EMA Questionnaire

An overview of the EMA protocol can be found in Supporting Information S1: List S4. Patients were asked to fill out the EMA questionnaire three times a day during a period of 6 weeks, 126 times in total. As there is no gold standard for an EMA protocol, our protocol was based on consultation with an expert and previously used protocols as found in the literature [29]. Text messages, containing a weblink to the electronic questionnaire, were sent at scheduled semi‐random times; a possible “starting time” was set, depending on the participant's sleep pattern. The day was divided into three 4.5 h blocks during which a message was sent. Minimum time between two messages was 1 h. Patients had 45 min to fill out the questionnaire. If the questionnaire had not been completed within 15 min, a reminder was sent. After 45 min, the link was closed. The actual fill‐in time was between 5 and 10 min.

The EMA Questionnaire consisted of 22 questions (Supporting Information S1: List S2). The EMA questionnaire for the current study was developed using items previously used in clinical research and archived in EMA questionnaire banks and adjectives to assess positive affect and negative affect were selected based on this previous research and discussions in our research team [20]. In the context of the current study, neither patients nor clinicians were involved in the development of the EMA. The first part consisting of 19 questions focused on the mood of the patient. Patients were asked to state how much the feelings were applicable to their mood, by using a visual‐analog scale. These visual‐analog scale were sliders, using a scale of 0–100 ranging from *“not at all”* till *“very much”*. Eleven of the nineteen mood questions could be clustered into two categories: “negative affect (NA)” and “positive affect (PA)”. Examples of these were: “at this moment I feel cheerfull (PA)” or “at this moment I feel anxious (NA)”. The other eight were questions on for example cognitions and stress: “today was so far stressful”. Questions were related to feelings often co‐occurring with depression such as feeling lonely, annoyed, helpless, guilty, satisfied, and empty. The final three questions addressed the current activity, its energy cost, and enjoyment, with an open answer space for comments provided at the end.

Weekly feedback reports, generated by Roqua, with graphical summaries of PA and NA scores were emailed to the patients.

At study completion, mean affect scores overall and per activity were calculated for each patient and included in a final report which was sent to the patients, with graphical trends over time. As this was a feasibility study focused on the procedures, healthcare providers did not receive feedback reports from the EMA data.

#### 2.5.3 EMA Evaluation

At the end of the study, patients were asked to fill out a self‐developed evaluation questionnaire containing 25 questions on the study and use of EMA. Questions were for example focused on the experienced length of the study, the logistics and the content of the EMA questions (see Supporting Information S1: List S3 for the evaluation questionnaire). Patients were also contacted by phone for feedback on their EMA experience and if they had any further feedback. The goal of the evaluation was to get more details on the feasibility of using EMA and to receive feedback for improvement.

#### 2.5.4 EPF Data

After completing the study, the following data were retrieved from the patient's Electronic Patient File (EPF): diagnosis (type of cancer, treatment, time between treatment and start study), comorbidity (somatic and psychiatric) and medication use. Data on care utilization were collected by capturing the frequency and reasons for hospital visits (follow‐up cancer treatment) and/or general practice during the study period and 1 month after. Comorbidity data were used to calculate the Charlson comorbidity index [30].

### 2.6 Ethical and Safety Issues

We addressed patient safety and well‐being as follows.

At recruitment, patients with major depressive disorder requiring immediate treatment (e.g., due to suicidality), were excluded and referred to the GP. During inclusion, patients were instructed to contact their GP if they experienced any concerns. At screening, a positive IDS suicide item triggered GP notification and implementation of a suicide prevention protocol. Throughout the study, patients were able to contact the study team by email with questions or concerns, for example after receiving the weekly reports. Patients were encouraged to contact their GP if they were worried about their mood. EMA data were not real‐time checked for alarming trends, but only reviewed at the end of the study. Patients were instructed to contact their GP in case they did not feel well. If at any time, signs of any suicidal thoughts were received (e.g., by email), the suicide prevention protocol was followed (patients and their GP were contacted).

### 2.7 Outcome Measurements

Our pilot study was focused on investigating the feasibility of using EMA in primary care for getting insight in the mood patterns associated with depression in cancer survivors. Feasibility was evaluated in terms of “reach and response”, “dose delivered”, “dose received”, and patient's satisfaction. Reach was defined as the number of patients meeting the inclusion criteria and not meeting the exclusion criteria, divided by the patients that were considered possibly eligible based on the EPF search. Response was defined as the proportion of patients that were willing to participate out of the number invited. After the first week of the study, we checked whether the text messages, containing weblinks, were actually received by the patients to see whether there were any technical issues (“Dose delivered”). “Dose received” was the number of EMA‐questionnaires that were filled out by the patients. Only if all questions on the questionnaire were filled in, this was considered as a “dose received” (completion). The number of reminders for filling out the questionnaire was evaluated as well.

Patient's satisfaction was measured through the evaluation questionnaire and the evaluation phone calls (see details above).

Mean levels of each of the measured PA and NA experienced were collected.

### 2.8 Data Analysis

Descriptive statistics were calculated for patient characteristics. For continuous variables, the mean and standard deviation were used to describe the data, if normally distributed. If data were not normally distributed, the median and interquartile range were presented. Percentages were calculated for categorical data. To describe the positive and negative affective experiences, we calculated the mean and standard deviation of each of the PA and NA items at the person level for the total study period. Data was analyzed using Stata version 17.0 and R version 4.2.0. Two‐sided *p*‐values < 0.05 were considered statistically significant.

## 3 Results

### 3.1 Recruitment

**FIGURE 1:**
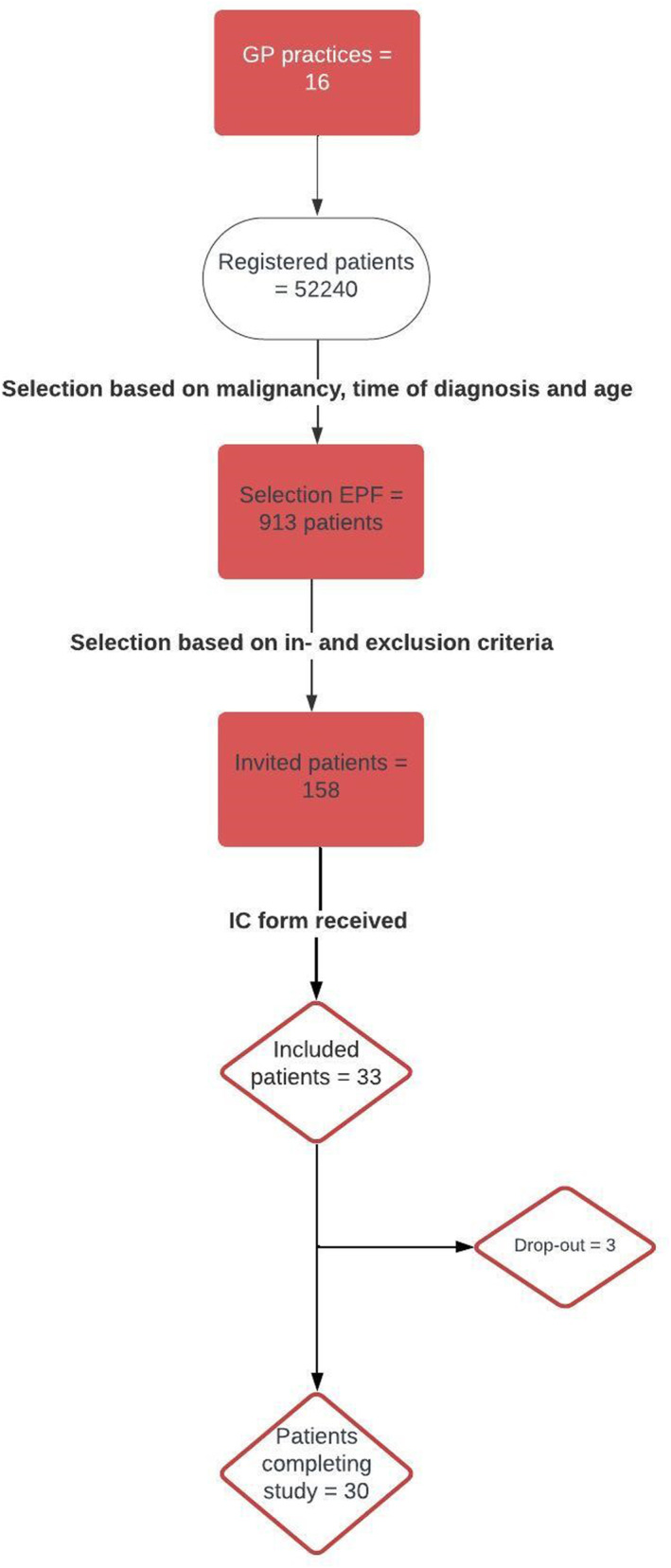
Flowchart of recruitment process. EPF = electronic patient file, IC = informed consent.

We included sixteen practices. One practice performed their own search, not leading to any included patients. The remaining fifteen practices included roughly 52240 registered patients. In total, 931 patients were possibly eligible based on the first EPF search (see Figure 1 for the flow chart). Of these, 158 patients were actually eligible, resulting in a reach of 17.0% (158/931). The most common reasons for exclusion were initial treatment more than 2 years ago and a palliative care setting (meaning no curative treatment for the malignancy). Of the 158 invited patients, 33 agreed to participate, yielding a response rate of 21.5%. When no response was received, patients were phoned to assess participation interest if possible. 36 patients were called. Most common reasons for not participating were: not interested (47%), not having any mood problems (even though this was not necessary, this made them feel unsuitable) (17%) and being too busy (14%). Except for the information given during the phone calls, no other data was available on the patients that did not want to participate, hence no comparison between the groups could be made.

### 3.2 Study Population

Three patients withdrew from the study: one due to mismatched expectations (withdrew before starting), one due to competing activities, and one due to anxiety caused by the text messages. The patient who withdrew immediately after inclusion did not fill out the baseline questionnaire. Table 1 shows the baseline characteristics. About half of the patients were female, the median age was 67 years (29–76). Most participants were in a relationship and had children. Most common diagnoses were breast, prostate and colon cancer.

**TABLE 1:** Baseline characteristics of the study population.

	*N* = 33
Age in years (median, range)	67 (29–76)
Female sex, *n*/*N* (%)	17/33 (52)
Educational level, *n*/*N* (%),	
Low	11/32 (34)
Average	8/32 (25)
High	13/32 (41)
Employed, *n*/*N* (%)	10/32 (31)
Living situation, *n*/*N* (%)	
Independent	31/32 (97)
Independent, with home care	1/32 (3)
Has partner, *n*/*N* (%)	25/32 (78)
Has children, *N* (%)	25/32 (78)
Social support, *n*/*N* (%)	
None	0/32 (0)
Low	4/32 (13)
Moderate	20/32 (63)
High	8/32 (25)
More than one cancer diagnosis, *n*/*N* (%)	7/33 (21)
Cancer diagnosis, *n*/*N* (%)	
Breast cancer	10/33 (30)
Prostate cancer	7/33 (21)
Colon cancer	7/33 (21)
Other	18/33 (56)
Time since most recent cancer diagnosis in months (median, range)	24.9 (3.5–166.6)
Charlson comorbidity index (median, range)	4 (2–7)
History of psychiatric comorbidity, *n*/*N* (%)	
Anxiety	1/33 (3)
Depressed mood	3/33 (9)
Depressed mood and anxiety	1/33 (3)
PTSS	2/33 (6)
None	25/33 (79)
Use of antidepressants during study period, *n*/*N* (%)	6/33 (18)
Use of benzodiazepines, *n*/*N* (%)	1/33 (3)
Consultation frequency—GP^a^ (median, range, *N* = 33)	1 (0–6)
Psychiatric reasons (*N* = 5)	1 (1–2)
Other reasons (*N* = 16)	2 (1–6)
Consultation frequency‐ hospital^a^ (median, range, *N* = 33)	1 (0–6)
Follow‐up cancer	0 (0–4)
Other	0 (0–5)

^a^ During the study period and 1 month after.

### 3.3 EMA Results

#### 3.3.1 Adherence to EMA

All EMA prompts sent were received by the participants ensuring a dose delivered of 100%. All participants received the same number of prompts. Three patients (9%) dropped out during the study (details given above). Of the 30 patients who completed the full study period, mean compliance to EMA questions was 67% (SD = 29). Four patients (13%) responded to fewer than 30% of the EMA prompts and were excluded from the remaining results on the EMA items. Mean compliance of the remaining 26 patients was 84% (SD = 20).

#### 3.3.2 Summary Statistics Positive and Negative Affective Experiences

Table 2 gives the mean values of the EMA items during the whole study period on the person level. Across all patients, the mean level of positive affective experiences was between 66 and 76 of maximal 100 and the mean level of negative affective experiences was between 9 and 17 of maximal 100 (see Table 2). For example, there were only three out of twenty‐six patients with low average levels of cheerfulness (i.e., having a mean level on cheerfulness lower than 50). The most prominent depressive symptom in this sample was a lack of energy. Mean levels of feeling energetic were 66 on a scale from 0 till 100 across patients and 27% of the sample (*n* = 7) reported on average low levels of feeling energetic (i.e., having a mean level on energy lower than 50).

**TABLE 2:** Summary statistics of responses to the EMA items.

Positive affect	Person mean of each EMA item			
	Mean (SD)	Minimum	Maximum	% (*n*) > 50^a^
Cheerful	73 (16)	45	96	88% (23)
Enthusiastic	70 (17)	45	97	85% (22)
Energetic	66 (22)	15	98	73% (19)
Content	76 (14)	50	98	96% (25)
Negative affect				
Fearful	10 (13)	0	47	0% (0)
Nervous	15 (18)	1	67	4% (1)
Lonely	16 (24)	0	97	12% (3)
Irritated	12 (13)	0	47	0% (0)
Helpless	10 (14)	0	57	4% (1)
Guilty	9 (12)	0	43	0% (0)
Empty	17 (17)	0	54	8% (2)

*Note: n* = 26. Range of scores = 0 to 100. Person mean = the average across all observations of one item of one individual. SD = standard deviation.

^a^ % (*n*) > 50 = the percentage of individuals scoring on average higher than 50 on a scale ranging from 0 (not at all) till 100 (very much).

#### 3.3.3 Feedback Reports

**FIGURE 2:**
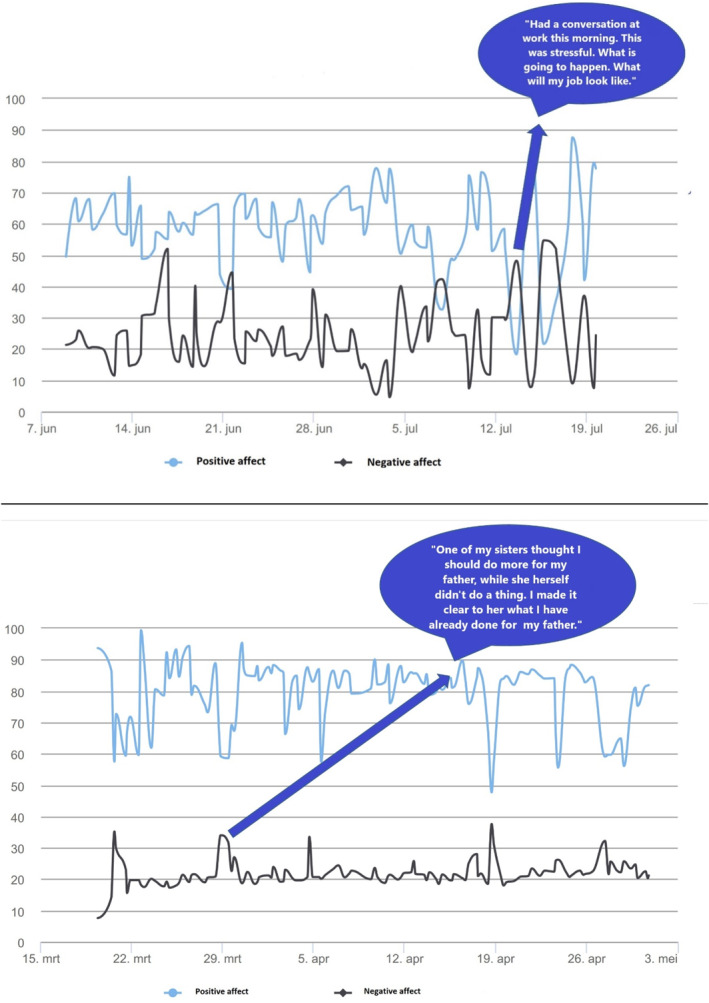
Two examples of the final report showing positive and negative affect scores and filled out quotes visualized in speech bubbles.

Figure 2 shows examples of feedback reports demonstrating the variance in PA and NA scores over a period of 6 weeks. Some patients filled out notes/free texts at the end of the EMA questionnaires (see speech bubbles), giving insight in reasons for changes in mood.

### 3.4 Patient's Satisfaction

Overall, 27 (82%) patients filled out the evaluation questionnaire (Table 3). Most patients agreed the study was easy to complete (67%). The majority indicated that the EMA questions were clear (75%) and that the weekly reports gave insight into their mood (75%). 63% of patients considered the frequency of EMA prompts per day too high, and 48% thought the 6 week period was too long.

**TABLE 3:** Patients' responses on a selection of questions from the evaluation questionnaire.

	*N* = 27, *N* (%)
Easy to complete the study	
Don't agree at all	1 (4)
Disagree a little	6 (22)
Neutral	2 (7)
Agree a little	8 (30)
Completely agree	10 (37)
EMA questions were clear	
Don't agree at all	0 (0)
Disagree a little	2 (7)
Neutral	4 (15)
Agree a little	6 (22)
Completely agree	15 (56)
The weekly reports gave insight in my mood	
Don't agree at all	0 (0)
Disagree a little	0 (0)
Neutral	7 (26)
Agree a little	11 (41)
Completely agree	9 (33)
Filling out questionnaires three times a day was:	
Way too little	0 (0)
Little too much	1 (4)
Exactly right amount	9 (33)
A bit too much	16 (59)
Way too much	1 (4)
Filling out the questionnaires during a period of 6 week was:	
Way too little	0 (0)
Little too much	0 (0)
Exactly right amount	14 (52)
A bit too much	11 (41)
Way too much	2 (7)

During the evaluation call most patients reported growing negligent and non‐compliant with filling out the questionnaires toward the end of the study. They attributed this to perceiving minimal changes in their mood and the identical order of questions. Other frequently mentioned room for improvement items were in the timing of the EMA prompts and the frequency of the text messages, because the messages were interfering with daily activities. Positive feedback included the weekly reports: they recognized themselves and thought the reports gave insight in their mood. One participant thought the report would be useful during consultations with the General Practice Mental Health Professional (GP‐MHP). Most patients did not have any trouble filling out the EMA questionnaires.

## 4 Discussion

In this pilot study we investigated the feasibility and patient's satisfaction of using EMA for detecting affect patterns associated with depression in cancer survivors in primary care. Our study showed that using EMA as in this study may not be entirely feasible, based on the reach and response rates, which were 17.1% and 20.8% respectively. However, for most of those who participated, completing the EMA questionnaires seemed feasible with an average completion rate of 67%. Only 4 participants responded to less than 30% of all EMA prompts. Furthermore, drop‐out rate was low (15%). The frequency of the questionnaires and the study duration were frequently considered a bit too much and a bit too long. Yet, patients found EMA user‐friendly and the weekly reports insightful regarding their mood.

### 4.1 Comparison With Existing Literature

EMA is increasingly used for detecting affective experiences related to several conditions, particularly in secondary care [31, 32, 33]. Several studies have explored EMA in cancer survivors in secondary and tertiary care [18, 26, 34]. A scoping review including twelve studies concluded that EMA, primarily focused on cancer‐related fatigue, is feasible and useful for examining real‐time emotions and behavior related to cancer and its treatment [35]. Another study on EMA feasibility in patients with chronic cancer‐related fatigue awaiting psychological care reported overall good patient satisfaction, though some participants, similar to our findings, noted a tendency to complete questionnaires “on the automatic pilot” [18]. Nevertheless, the authors of this study concluded that EMA seemed feasible and useful for giving insight in experienced complaints, such as fatigue or psychological problems, which could be used during psycho‐oncological treatment. These results are in line with ours considering the insight that EMA may provide into mood symptoms. However, other studies have reported higher questionnaire completion rates of 80% or more [18, 35, 36]. To our knowledge, no other studies have investigated its use in primary care.

In our study participants considered EMA easy to use and regarded the weekly reports as providing insight into their mood. Our different conclusion regarding feasibility, could be explained by several factors, in particular study population and setting: most of our participants did not experience any mental problems, as far as we know, and this could hamper motivation. The EMA questions were focused on experiences related to depression that may not have been considered relevant by the participants. Thus, EMA could be more feasible in populations with known depressive symptoms. They might benefit from receiving feedback on their EMA scores, which they can discuss with their physician.

The primary care setting of our study could have impacted the feasibility as well: patients were invited to participate by letter, as they often don't visit the GP that much. If they would have been invited in person by a physician they see more often, they might me more motivated to participate.

An interesting finding within our study was the on average low scores on the question on feeling energetic. This could be a sign of cancer‐related fatigue. Previous research indicates that up to 35% of patients treated for lymphoma, breast, cervical and testicular disease suffers from chronic fatigue [37]. In our study the EMA questions were not focused on cancer‐related fatigue, but the item “feeling energetic” could serve as an alternative way for assessing fatigue leveraging EMA's advantages, in particular addressing cognitive problems in cancer survivors.

### 4.2 Strengths and Limitations

An important limitation of our study was that participants were not selected on the presence of depressive symptoms at baseline. Initially a score of two or higher on the Patient Health Questionnaire‐2 (PHQ‐2) was an inclusion criterion, as it aligns with the NHG depression guideline 2024 [38] and clinical practice for the monitoring of depression [39]. However, by far most patients who were willing to participate had a score of zero on the PHQ, whereas we expected based on previous research depressive symptoms prevalence rates between 12% and 20% in our target population [1]. Only four patients met this inclusion criterium; three patients had a score of two and one patient had a score of three. Consequently, we decided to remove this inclusion criterion. The low prevalence of depressive symptoms in our sample could in part be the result of self‐selection bias, as patients with fewer or less severe depressive symptoms may be more inclined to participate than those with more depressive symptoms [40]. Therefore, our results may not be directly generalizable to clinical (research) settings.

Another limitation may have been the identical order in which questions were asked. Feedback from patients included that this led to negligence and monotone scores for each prompt.

Another limitation of our study was that, given its focus on feasibility, no direct feedback from the EMA data was given to the GP to be used to improve patient care. For that reason, GPs and patients might not see much benefit in participating and could have made them less eager to participate. Finally, the strict operationalization of initial treatment and the study‐design–based exclusion of patients treated for depressive symptoms may partly explain the reduced reach and a potential underestimation of EMA feasibility in primary care for oncological patients.

A strength of our study was the quantity and quality of feedback that we got from the participants from our evaluation questionnaires and phone calls on the feasibility and satisfaction of using EMA. A limitation was that we did not use any standardized tool for the evaluation.

In addition, we noticed that many participants used the final “open answer” box in the EMA questionnaire. This gave us a broader insight on one's mood and more context of the setting. In previous studies this broad insight on experienced symptoms and context specificity of symptoms were considered useful in clinical practice by clinicians [41, 42].

### 4.3 Implications for Research and Practice

The feasibility of EMA may be further investigated in primary care in patients visiting the GP or GP‐MHP with depressive symptoms. For those patients EMA might provide insight into mood, which could be of use during consultations with the GP or GP‐MHP as well, extending beyond cancer survivors. Based on our results, we would suggest the frequency and duration of EMA could both be decreased, to improve feasibility. The protocol could be adapted to the individual needs of the patients and health care professional. Our study showed that the reach and response rates do not provide sufficient support recommendation EMA as a tool for cancer survivors without manifest depressive symptoms in primary care. However, EMA seems feasible for those who are willing to try EMA and this may provide insight into their mood. This may especially useful for cancer survivors who are experiencing depressive symptoms. Furthermore, research indicated that specifically for cancer survivors, EMA could be particularly useful in tracking cancer‐related fatigue patterns, potentially identifying triggers affecting fatigue levels. Currently, a study is being performed amongst cancer survivors in secondary care in which EMA's therapeutic efficacy is being investigated [43]. The focus is on fear of recurrence, cancer‐related fatigue and depressive symptoms. The findings could be used for further research within primary care.

The initial thought of using EMA as a tool was to help with early recognition of depressive symptoms; however, our study showed that EMA was not feasible in this setting, partially due to participants not having any symptoms that they were aware of. Due to the pilot setting and focus on feasibility of our study, we could not investigate whether EMA was able to timely detect affect patterns associated with depression. Further research is needed to explore this further.

## 5 Conclusion

Overall, we conclude that using EMA is at present not entirely feasible to offer as a tool to gain insight in mood in cancer survivors within primary care without signs of depression, based on the low reach and response rates. However, EMA was considered easy to use as a tool and provided useful insights to those motivated to engage in EMA. EMA may be useful for cancer survivors and other primary care patients with depressive symptoms.

## Funding

This study was supported by a grant from Stichting Stoffels‐Hornstra (2019).

## Ethics Statement

A waiver for medical ethical approval was obtained from the Medical Ethical Review Board of the University Medical Center Groningen, METc 2020/348; register number 20200464.

## Conflicts of Interest

The authors declare no conflicts of interest.

## Supporting information


Supporting Information S1

## Data Availability

The data that support the findings of this study are available from the corresponding author upon reasonable request. [dataset] Panjer JA, Stegmann ME, Brandenbarg D, Schroevers MJ, Riese H, Snippe E, Burger, H; 2025; Ecological Momentary Assessment data from cancer survivors from primary care. Data set. Available upon request from Department of Primary and Long‐term care, University Medical Center Groningen (h.burger@umcg.nl).
