# Supplementary material for: Ecological Momentary Assessment for Assessing Affect Patterns Associated With Depression in Cancer Survivors in Primary Care: A Pilot Study
Source: Psychooncology. 2025 Dec 31;35(1):e70367. doi: 10.1002/pon.70367 (PMC12755119; doi:10.1002/pon.70367)
Supplement: Supplementary file 1 — Supporting Information S1 [file PON-35-e70367-s001.docx]

**Supplements**

**S1** List of all included malignancies and their ICPC codes

- Colorectal carcinoma (D75)
- Prostate carcinoma (Y77)
- Breast carcinoma (X76)
  - Adeno carcinoma breast woman (X76.01)
- Hodgkin’s (B72.01)
- Malignancy stomach (D74)
- Malignancy pancreas (D76)
- Malignancy other digestive organs (D77)
  - Esophagus (D77.01)
  - Salivary glands (D77.02)
  - Lip/ tongue/ mouth (D77.03)
  - Liver/Gall bladder/ Bile ducts (D77.04)
- Malignancy eye (F74.01)
- Malignancy ear (H75.01)
- Malignancy musculoskeletal system (L71.01)
- Malignancy nervous system (N74)
- Malignancy bronchus/lung (R84)
- Malignant melanoma (S77.03)
- Malignancy thyroid (T71)
- Malignancy kidney (U75)
- Malignancy bladder (U76)
- Malignancy cervix (X75)
- Malignancy sexual organs man (Y78)
  - Penis (Y78.01)
  - Testis (Y78.02)
  - Breast (Y78.02)

**S2** EMA questionnaire

**Today was so far….**

(1) Enjoyable

0-100

(2) Stressful

0-100

**At this moment…**

(3) … I am worried

0-100

(4) … I am thinking about how I feel

0-100

(5) … I feel the urge to…

Let my thoughts in – avoid my thoughts

**What I have in mind is….**

(6) Negative- Positive

**At this moment I feel….**

(7) Happy-Sad
0-100

(8) … Cheerful

0-100

(9) … Anxious

0-100

(10) … Nervous

0-100

(11) … Enthusiastic

0-100

(12) … Lonely

0-100

(13) … Energetic

0-100

(14) … Irritated

0-100

(15) … Helpless

0-100

(16) … Guilty

0-100

(17) … Satisfied

0-100

(18) … Empty

0-100

**At this moment I feel the urge to….**

(19) … let my emotions in - … avoid my emotions

(20) Which of the options below describes the best what you did in the past half hour?

- Work / School
- Households / Groceries
- Eat / Drink
- Social contact
- Personal hygiene / put make-up
- Taking care of others
- Relaxing
- Sports
- Travel
- Sleep

**I enjoyed the activity I was doing**

(21) Not at all – Very much

**The activity I was doing cost me energy**

(22) Not at all – Very much

**Open Q: Write down below if you have anything else you would like to say. For example, you could describe your (un)pleasant experiences here, so you can recall them later:**

**Note:**

**Combination of questions leading to total score on positive affect:** 8, 11, 13, 17

**Combination of questions leading to total score on negative affect:** 9, 10, 12, 14, 15, 16, 18

**S3 Evaluation questionnaire**

*All questions are answered on a five-point scale from don’t agree at all to completely agree.*

- I found the way in which the project researchers approached me pleasant
- The information flyer for the project was informative
- The information flyer for the project was too long (too short, just right, too long categories)
- Contact with the researchers was experienced as pleasant
- The researchers have sufficiently explained the importance of the research
- The researchers took enough time for all my questions or uncertainties (also n/a option)
- If I needed help from the researchers, they were easily accessible (also n/a option)
- The researchers helped me a lot with technical problems (also n/a option)
- I found it easy to complete the diaries
- I thought the questions in the diaries were clear
- I found the questions in the diaries confronting
- I liked the questions in the diaries
- I found the questions in the diaries valuable
- Completing the diaries has given me insight into my positive mood
- Completing the diaries has given me insight into my negative mood
- The weekly reports I received were informative
- I hated receiving a weekly report of my mood
- The final report was informative
- The final report was complete
- I thought answering the questions three times a day was just enough (scale too little to too much)
- I thought answering questions three time s a day for 6 weeks was too much (scale too little to too much)
- The GP was well informed about the project (also n/a option)
- The GP paid sufficient attention to my mood complaints (also n/a option)
- I was able to help my fellow patients by participating in this study

*Note: ‘diaries’ is referring to the EMA questionnaires.*

**S4 EMA protocol**

| **Start EMA protocol** | Personalized starting time can be set  Day divided in 3 blocks of 4,5 hours after starting time  EMA questionnaire sent at random  3 times a day, for 6 weeks |
| --- | --- |
| **EMA questionnaire logistics** | Text message sent with weblink to questionnaire  Reminder sent after 15 minutes  45 minutes to fill in the questionnaire after first text message  Minimum of 1 hour between two questionnaires sent |
| **EMA questionnaire** | 22 questions  19 questions on mood, scale (0-100)  3 questions on current activity, energy cost and enjoyment  Open answer box |
